# Supplementary material for: Long-Term Exposure to Supraphysiological Levels of Testosterone Impacts Rat Submandibular Gland Proteome
Source: Int J Mol Sci. 2023 Dec 31;25(1):550. doi: 10.3390/ijms25010550 (PMC10778877; doi:10.3390/ijms25010550)
Supplement: Supplementary file 1 [file ijms-25-00550-s001.zip › Figures S1-S4.pdf]

Supplementary Materials

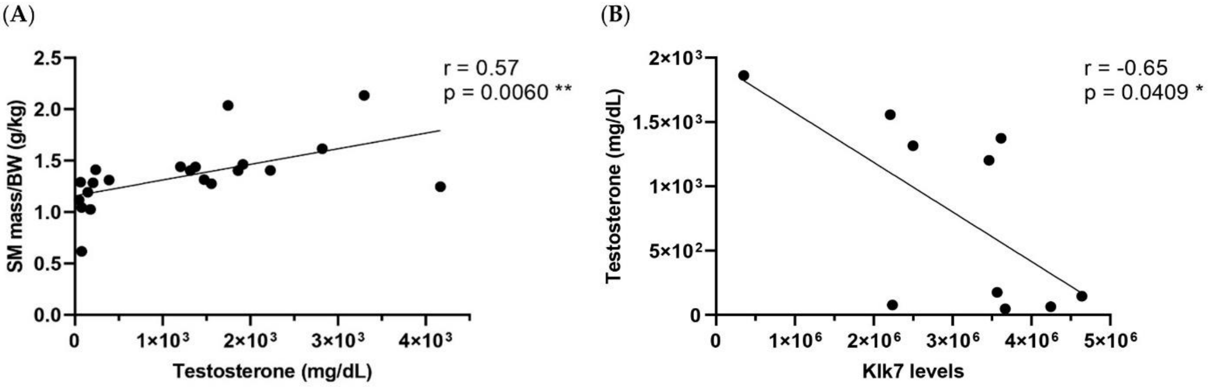

Figure S1. Pearson correlations were performed for an in-deep understanding of association between submandibular gland mass-to-body weight and testosterone levels (**A**), and testosterone and Klk7 (**B**). (* $p < 0.05$; ** $p < 0.01$).

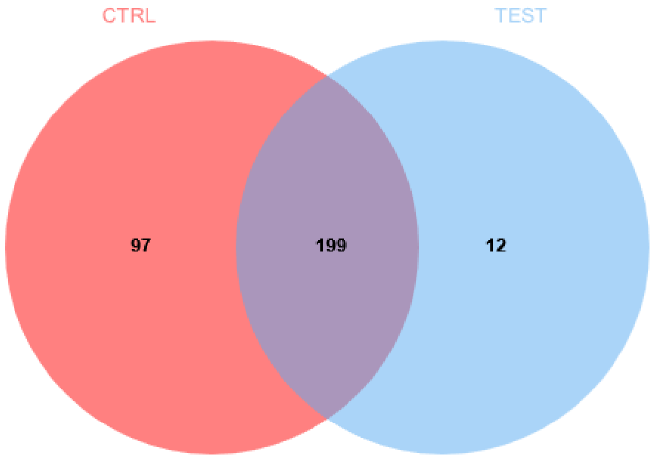

Figure S2. Venn diagram illustrating common proteins among CTRL and TEST groups, and exclusive proteins from each group. The represented figure was made with *jvenn* tool (jvenn.toulouse.inrae.fr).

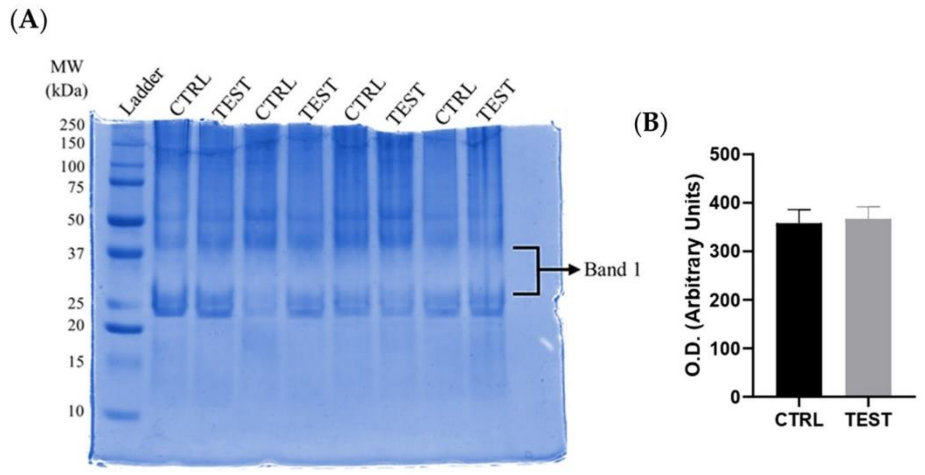

Figure S3. Zymography analysis of submandibular gland samples from CTRL and TEST. (**A**) Representative zymography gel of alternated submandibular gland samples from CTRL group and TEST group. A representative molecular weight standard (ladder) is shown. The band identified with proteolytic activity can be observed in the molecular weight range between ≈ 25 kDa and ≈ 40 kDa. (**B**) Optical density measurements of the referred samples obtained from *Image Lab* (O.D. is presented in arbitrary units).

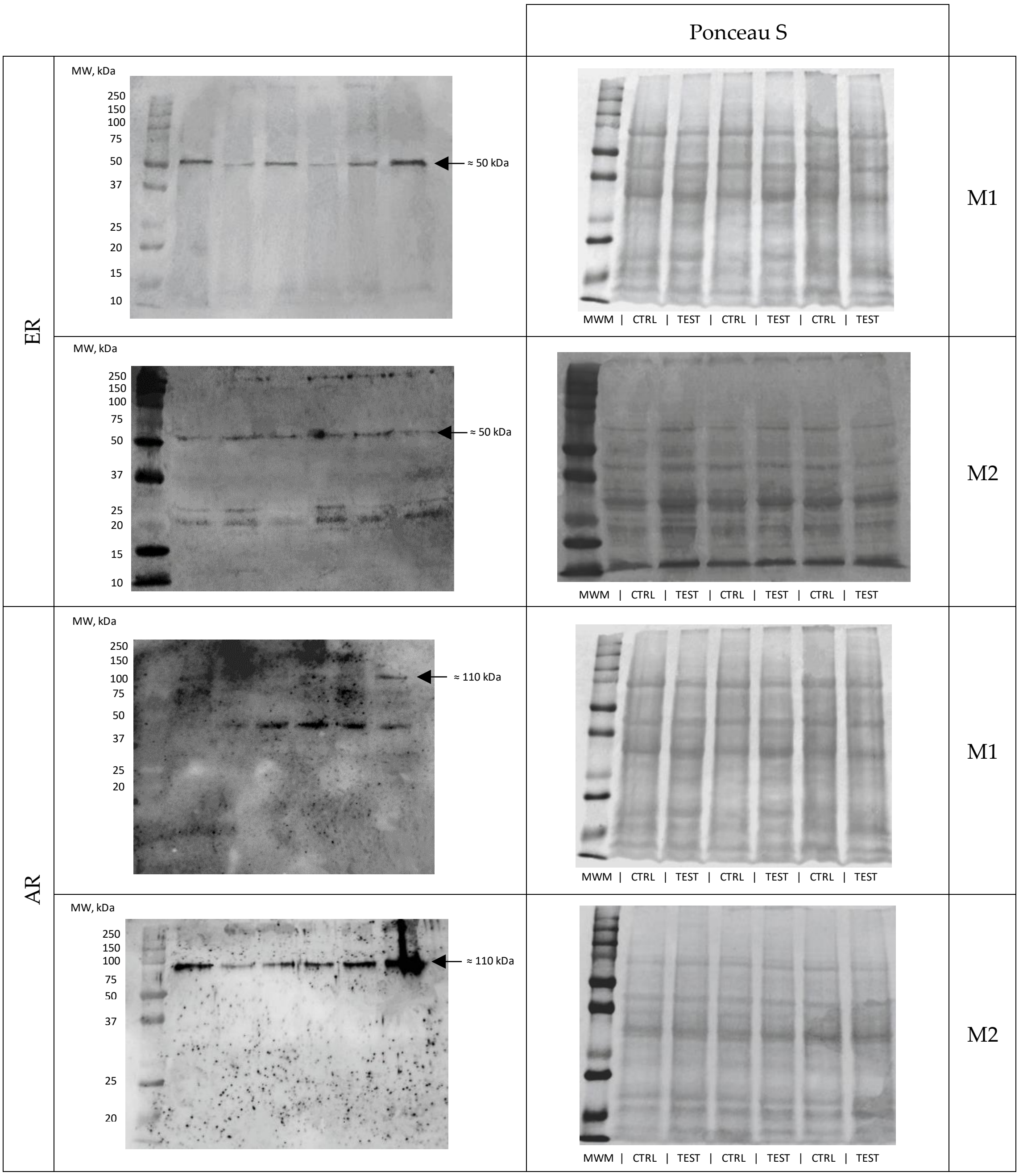

Figure S4. Ponceau S-stained nitrocellulose membranes and corresponding immunoblots of the proteins evaluated. MW, Molecular Weight.
